# Transient receptor potential canonical 5 channels plays an essential role in hepatic dyslipidemia associated with cholestasis

**DOI:** 10.1038/s41598-017-02439-z

**Published:** 2017-05-24

**Authors:** Khadija M. Alawi, David Tandio, Jin Xu, Pratish Thakore, Georgia Papacleovoulou, Elizabeth S. Fernandes, Cristina Legido-Quigley, Catherine Williamson, Susan D. Brain

**Affiliations:** 1 BHF Cardiovascular Centre of Excellence and Centre of Integrative Biomedicine, Cardiovascular Division, King’s College London, London, UK; 2 Institute of Pharmaceutical Sciences, Faculty of Life Sciences & Medicine, King’s College London, London, UK; 3 Division of Women’s Health, Women’s Health Academic Centre, King’s College London, London, UK; 4 Programa de Pós-Graduação, Universidade Ceuma, São Luís, Brazil

## Abstract

Transient receptor potential canonical 5 (TRPC5), a calcium-permeable, non-selective cation channel is expressed in the periphery, but there is limited knowledge of its regulatory roles *in vivo*. Endogenous modulators of TRPC5 include a range of phospholipids that have an established role in liver disease, including lysophosphatidylcholine (LPC). Cholestasis is characterized by impairment of excretion of bile acids, leading to elevation of hepatic bile acids. We investigated the contribution of TRPC5 in a murine model of cholestasis. Wild-type (WT) and TRPC5 knock-out (KO) mice were fed a diet supplemented with 0.5% cholic acid (CA) for 21 days. CA-diet supplementation resulted in enlargement of the liver in WT mice, which was ameliorated in TRPC5 KO mice. Hepatic bile acid and lipid content was elevated in WT mice, with a reduction observed in TRPC5 KO mice. Consistently, liver enzymes were significantly increased in cholestatic WT mice and significantly blunted in TRPC5 KO mice. Localized dyslipidaemia, secondary to cholestasis, was investigated utilizing a selected lipid analysis. This revealed significant perturbations in the lipid profile following CA-diet feeding, with increased cholesterol, triglycerides and phospholipids, in WT, but not TRPC5 KO mice. Our results suggest that activation of TRPC5 contributes to the development of cholestasis and associated dyslipidemia. Modulation of TRPC5 activity may present as a novel therapeutic target for liver disease.

## Introduction

Mammalian transient receptor potential (TRP) channels encompass over 28 members^1^, and exert wide-ranging functions peripherally and centrally. These non-selective cation channels form a major class of Ca^2+^-permeable channels, and are classified into five further subfamilies, based on amino acid sequence homology^2^. TRPC5 is a member of the canonical subfamily (TRPC) which comprise six members in humans^3^. TRPC5 is widely expressed in the central nervous system^4^ and, to a lesser extent, in the periphery^2, 5, 6^. TRPC5 assembles as a homo- or hetero-tetramer (Clapham, 2003), commonly associating with TRPC1 and TRPC4^2^. Moreover, there is some knowledge of TRPC5 activity in vascular/cardiovascular regulation^7, 8^. TRPC5 is known to be modulated by dietary lipids^9^, in addition to endogenous lipids^10^, with a regulatory role identified for this channel in adipocytes and adipokine secretion^7, 9^. Indeed, a range of biological properties have been determined from cellular studies, but less so for intact systems. The use of WT and TRPC5 knockout (KO) mice have allowed an understanding of potential roles to be delineated *in vivo*. We recently demonstrated that TRPC5 regulates inflammatory joint pathology in studies involving WT and TRPC5 KO mice^11^. However, at present, little is known about its role in hepatic (patho)physiology.

TRPC1 and TRPC6 were identified to be functionally expressed in human liver, with a prominent role for TRPC6 in hepatic carcinoma, attributed to aberrant calcium signaling^12^; TRPC5 commonly associates with TRPC1^2^ and it is expressed at low levels in the liver^6^. Additionally, TRPC channels are known to be expressed in different vascular beds^13, 14^, including the hepatic system, where TRPC1/TRPC5 complexes were detected in the portal vein of rabbits and shown to act as a store-operated channel^15^.

Cholestatic liver disorders comprising primary biliary cirrhosis, intrahepatic cholestasis of pregnancy and familial intrahepatic cholestasis, result in focal and systemic pathology, and critically, liver failure^16^. These pathologies are characterised by an impairment of hepatocellular and/or cholangiocellular secretory function and bile flow, resulting in elevation of hepatic and serum bile acid content^17^. During cholestatic liver diseases, accumulation of bile acids not only disrupts bile acid homeostasis directly, but alters the expression of critical genes involved in lipid homeostasis resulting in retention of lipids in the liver, ultimately leading to cell death and inflammation^18^.

Cholic acid (CA) is increased in patients with liver disease, and rodent experimental models of cholestasis utilising exogenous CA, e.g. via dietary supplementation, lead to hypercholanemia, dyslipidaemia and cholestatic symptoms^19^. Despite the increased knowledge of bile acid signaling and homeostasis, through identification of bile acid receptors, the pathogenesis of cholestasis remains unclear, with limited drug treatments available. While elevated serum bile acids remain a diagnostic marker for cholestatic liver disease, there is increasing evidence for dysregulation of lipid homeostasis, resulting in elevation of hepatic and serum lipids/metabolites^17, 19^.

As there is increasing evidence that TRPC5 plays a role in lipid sensing, and that TRPC5 is modulated by endogenous lipids that have an established role in cholestasis^17^, we hypothesized that TRPC5 has a role in the pathogenesis of cholestasis.

## Results

### TRPC5 deletion protects mice from cholestasis-induced liver injury

**Figure 1:**
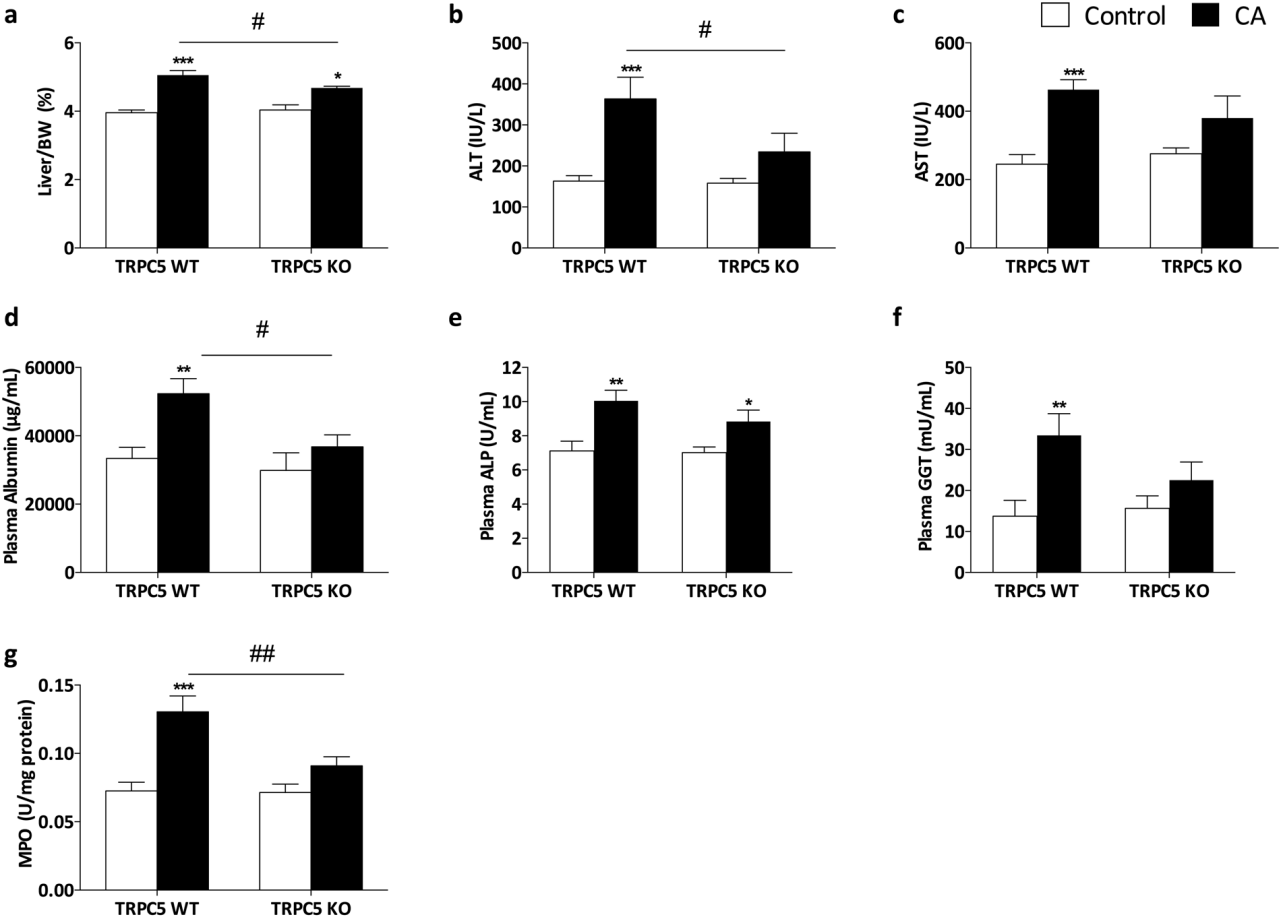
Cholestasis-induced liver injury is attenuated in TRPC5 KO mice. WT and TRPC5 KO mice were fed control (RM3) or CA (0.5% cholic acid supplemented RM3 diet) for 21 days. (**a**) Liver/body weight ratios in WT and TRPC5 KO mice. Liver injury was assessed by measuring plasma ALT (**b**), AST (**c**,**d**) albumin, (**e**) ALP, and (**f**) GGT. (**g**) Hepatic myeloperoxidase (MPO) activity following CA-induced cholestasis in WT and TRPC5 KO mice. Results are mean ± S.E.M., n = 6–7. *P < 0.05, ***P < 0.001 vs control; ^#^P < 0.05 vs WT CA as determined by two-way ANOVA and Bonferronni Post Hoc tests.

Consistent with previous reports^19, 20^, CA supplementation of WT animals caused significant liver injury, characterised by increased serum alanine transaminase (ALT) and aspartate aminotransferase (AST) levels, in addition to elevated liver weight (P < 0.01; Fig. 1a). Deletion of TRPC5 resulted in a marked attenuation in hepatomegaly when compared to WT mice (P < 0.05). Liver enzymes (ALT, AST, ALP and GGT) were consistently elevated in CA-fed WT mice (Fig. 1b–f) compared to chow-fed mice (P < 0.001), and attenuated in TRPC5 KO mice. In line with this, plasma albumin concentrations (Fig. 1d) was significantly increased in WT mice following CA-diet feeding with a significant reduction in TRPC5 KO mice (P < 0.05). We measured hepatic myeloperoxidase (MPO) activity, a marker of inflammatory leukocyte accumulation^21, 22^, and found a significant increase in liver samples of WT CA-fed mice (P < 0.001). This increase in MPO activity was significantly diminished in TRPC5 KO mice (P < 0.01; Fig. 1e), indicative of reduced inflammation in these animals. Both groups of mice demonstrated slight weight loss following CA-diet feeding (Supplementary Fig. 1A), as previously documented^23^. However, the extent of the reduction was not significantly different between groups (Supplementary Fig. 1B). This reduction in body weight was not associated with reduced food/water consumption (Supplementary Fig. 1C,D), and blood pressure was not altered between groups (Supplementary Fig. 1E).

### TRPC5 deletion protects mice from hypercholanemia and liver injury

**Figure 2:**
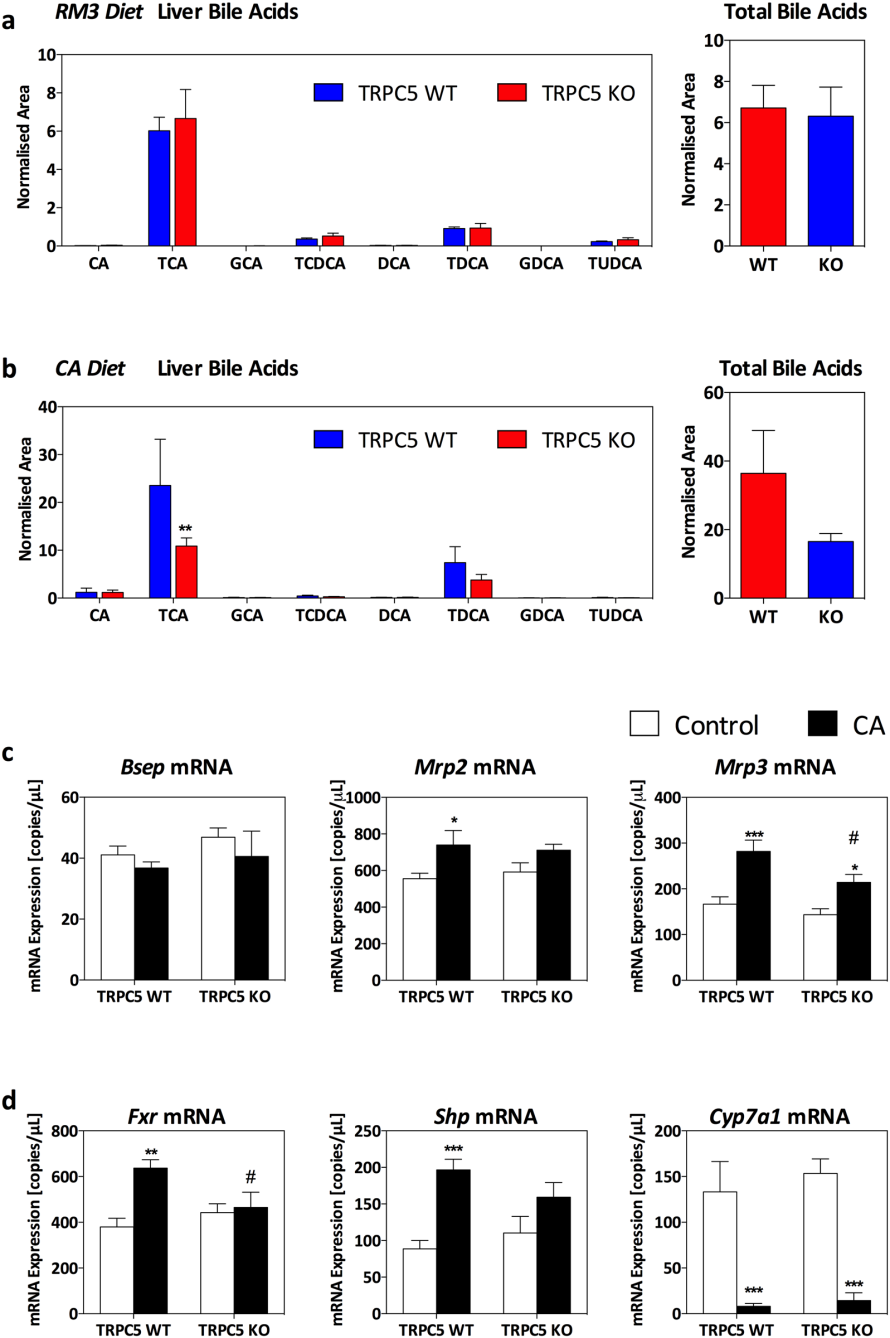
Deletion of TRPC5 attenuates hepatic hypercholanemia. WT and TRPC5 KO mice were fed control (RM3) or CA (0.5% cholic acid supplemented RM3 diet) for 21 days, and (**a**) bile acids were measured in RM3 control diet fed mice and (**b**) CA-diet with respective total bile acids (normalized area). (**c**) Hepatic gene expression determined by real-time quantitative PCR of bile acid transporters, bile salt export pump (Bsep), multidrug resistance-associated protein 2 (Mrp2), and multidrug resistance-associated protein 3 (Mrp3), and bile acid homeostasis/regulatory genes (**d**), farsenoid-X-receptor (Fxr), small heterodimer partner (Shp), and cytochrome P450 family 7 subfamily A member 1 (Cyp7a1), normalized to HPRT, β-actin, and B_2_M. Results are mean ± S.E.M., n = 6–7. *P < 0.05, **P < 0.01, ***P < 0.001 vs control; ^#^P < 0.05 vs WT CA as determined by two-way ANOVA and Bonferronni Post Hoc tests.

Hepatic BAs were measured by ultra-performance liquid chromatography-mass spectrometry (UPLC-MS) and demonstrated an increase in taurocholic acid (TCA) in WT CA mice which was significantly attenuated in TRPC5 KO mice (Fig. 2b; P < 0.01). Collectively, primary and secondary BAs were increased, and total BA levels were higher in CA-fed WT and TRPC5 KO mice compared to control-fed mice (Fig. 2a,b). Elevated BAs result in activation of the nuclear bile acid receptor, farsenoid-X-receptor (FXR), which modulates bile flow by regulation of its target genes, including canalicular transporters^24^. Accordingly, hepatic gene expression of key bile acid transporters, Bsep, Mrp2 and Mrp3 (Fig. 2c) demonstrated a significant induction in WT CA-fed mice. In contrast, induction of these transporters were significantly abrogated in TRPC5 KO mice.

In addition to regulating the expression of bile acid transporters, FXR is involved in bile acid homeostasis by regulating the activity of BA biosynthesis enzymes^25^. We did not observe differences in the hepatic expression of FXR (Fig. 2d); however, a significant induction in the expression of its key target gene, small heterodimer partner (Shp), was demonstrated in WT CA-fed mice (Fig. 2d), which did not reach significance in TRPC5 KO mice. FXR/SHP regulate bile acid biosynthesis through a characteristic repression of CYP7A1^26^, the rate-limiting enzyme in the conversion of bile acids from cholesterol. Consistent with this, we observed a significant repression of CYP7A1 in both WT and TRPC5 KO mice, suggesting that this negative feedback repression is independent of TRPC5. In line with this, analysis of plasma BAs plasma demonstrated hypercholanemia (Table 1), with a significant increase in plasma CA, TCA, and total BAs in CA-fed mice of both genotypes.

**Table 1:** Plasma Bile Acid profile.

Bile Acid (*NA*)	TRPC5 WT		TRPC5 KO	
	RM3	CA	RM3	CA
*CA*	0.15 ± 0.07	1.09 ± 0.36**	0.30 ± 0.07	0.88 ± 0.19
*TCA*	0.44 ± 0.22	1.30 ± 0.29*	0.33 ± 0.17	1.17 ± 0.2*
*GCA*	<LOQ	0.13 ± 0.03	<LOQ	0.10 ± 0.02
*TCDCA*	0.02 ± 0.01	0.04 ± 0.02	0.03 ± 0.01	0.03 ± 0.01
*DCA*	0.07 ± 0.03	0.94 ± 0.31	0.08 ± 0.02	1.33 ± 0.38
*TDCA*	0.23 ± 0.09	1.39 ± 0.23	0.08 ± 0.02	1.15 ± 0.38
*GDCA*	<LOQ	<LOQ	<LOQ	<LOQ
*TUDCA*	<LOQ	<LOQ	<LOQ	<LOQ
*TOTAL*	0.75 ± 0.26	4.92 ± 0.83**	0.82 ± 0.17	4.69 ± 0.95**

Primary and secondary plasma bile acids determined by LC-MS in WT and TRPC5 KO mice 21 days following chow- or CA-diet feeding. Results are mean ± S.E.M., n = 6–7. *P < 0.05, **P < 0.01 vs control as determined by two-way ANOVA and Bonferronni Post Hoc tests. CA; Cholic acid, TCA; Taurocholic acid, GCA; glycocholic acid, TCDCA; Taurochenodeoxycholic acid, DCA; Deoxycholic acid, TDCA; Taurodeoxycholic acid, GDCA, Glycodeoxycholic acid, TUDCA; Tauroursodeoxycholic acid. NA; normalized area, LOQ; limit of quantification.

### A role for TRPC5 in cholestasis-induced dyslipidaemia

**Figure 3:**
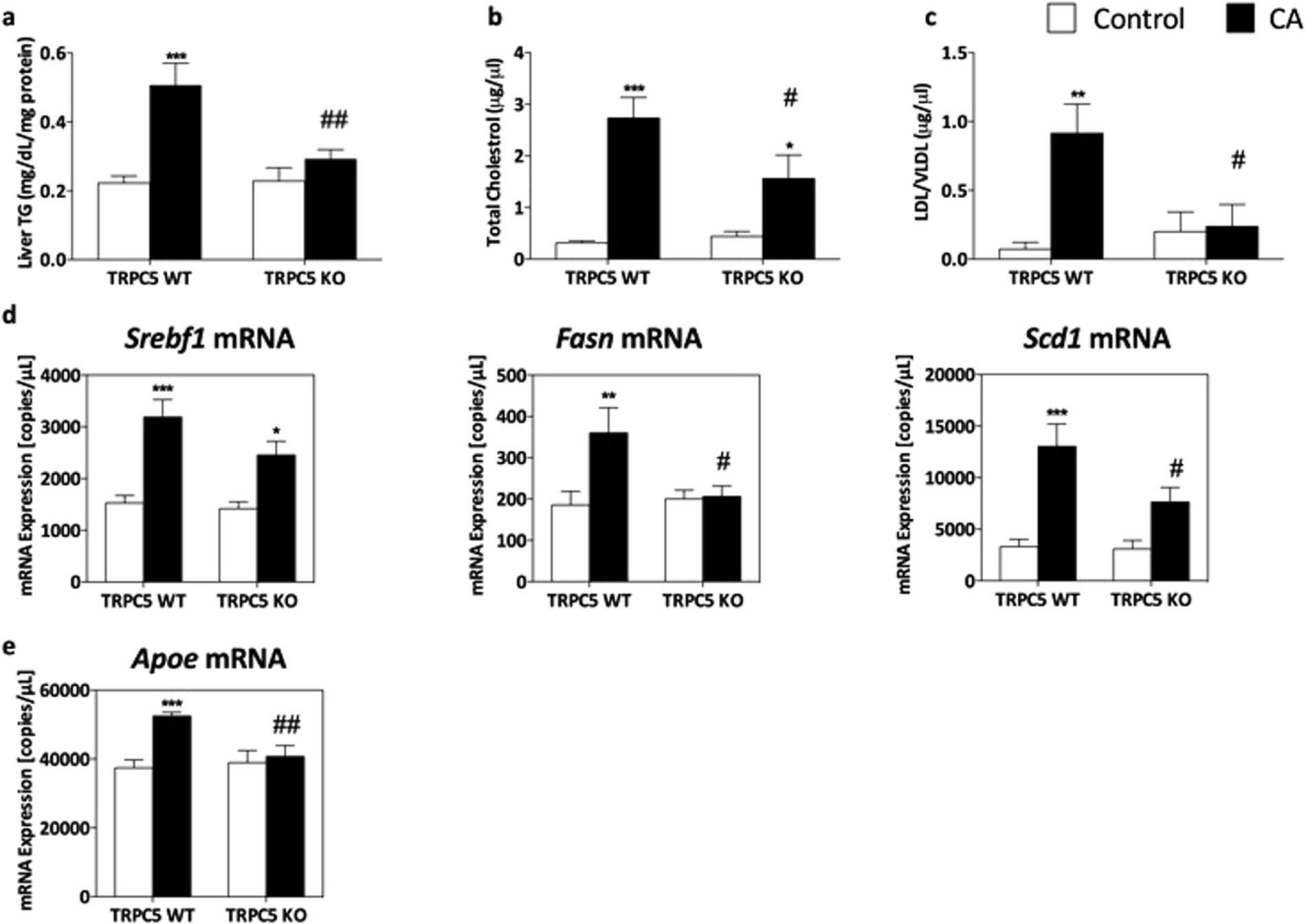
Cholestasis-induced hepatic dyslipidaemia is attenuated in TRPC5 KO mice. WT and TRPC5 KO mice were fed control (RM3) or CA (0.5% cholic acid supplemented RM3 diet) for 21 days, and (**a**) liver triglyceride (TG), total cholesterol (**b**), and LDL/VLDL (**c**) concentrations were measured. Hepatic gene expression determined by real-time quantitative PCR of genes involved in lipid biosynthesis (**d**), sterol regulatory element-binding transcription factor 1 (Srebf1), fatty acid synthase (Fasn), stearoyl-Coenzyme A desaturase 1 (Scd1) and (**e**) apolipoprotein E (Apoe), normalized to HPRT, β-actin, and B_2_M. Results are mean ± S.E.M., n = 6–7. *P < 0.05, ***P < 0.001 vs control; ^#^P < 0.05 vs WT CA as determined by two-way ANOVA and Bonferronni Post Hoc tests.

Cholestasis-associated hepatic steatosis^18^ was investigated, and while analysis of liver morphology by microscopy revealed little differences between control- and CA-fed mice, of both groups (Supplementary Fig. 3), we observed elevated hepatic triglycerides (TG), cholesterol and low-density/very low-density lipoprotein levels in WT mice following CA feeding (Fig. 3a–c), which were abborogated in TRPC5 KO mice; moreover, a significant difference was determined when compared to WT CA (P < 0.01; Fig. 3a). Total cholesterol was significantly increased in TRPC5 KO mice (P < 0.05); however, it was markedly attenuated compared to WT CA-fed mice (P < 0.05; Fig. 3b). This increase in hepatic lipid content was localized, as plasma TG levels were not significantly different between WT CA and control groups. Additionally, plasma cholesterol levels were unchanged in both WT and TRPC5 KO mice (Supplementary Fig. 2), although a modest reduction in WT CA-fed mice was observed (Supplementary Fig. 2), which may be downstream of CYP7A1 repression^27^.

We investigated the hepatic gene expression of mediators of lipid homeostasis and observed a significant induction in the expression of the major lipogenesis transcription factor, sterol regulatory element binding transcription factor 1 (Srebf1), and fatty acid synthase (Fasn) in WT CA groups compared to control (Fig. 3d). Additionally, the expression of stearoyl-CoA desaturase-1 (Scd1), which catalyses the synthesis of unsaturated fatty acids was significantly increased in WT CA-fed samples compared to chow-fed mice. The expression of apolipoprotein E (Apoe), involved in catabolism of TG, was significantly augmented in WT CA-fed compared to chow-fed mice (P < 0.001, Fig. 3e). The expression of these mediators were unchanged in TRPC5 KO CA mice and significantly blunted compared to WT CA mice.

### Lipid analysis and dysregulation of hepatic lysophospholipids

**Figure 4:**
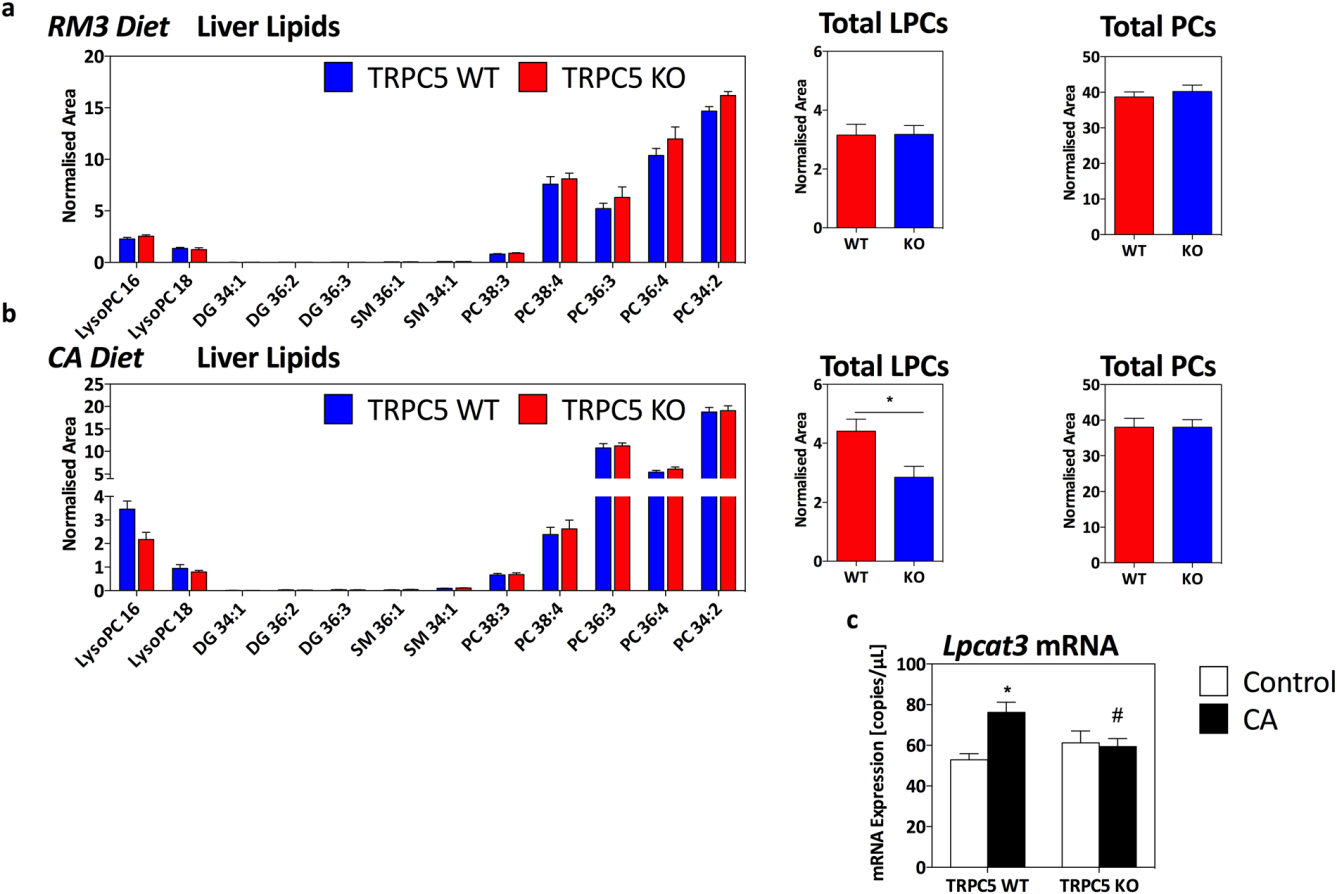
Hepatic lipid profile following CA-induced cholestasis. (**a**) Phospholipid species in WT and TRPC5 KO mice fed control (RM3) diet and (**b**), CA (0.5% cholic acid supplemented RM3 diet) for 21 days with respective total lysophosphatidylcholine and phosphatylcholine species (normalized area). (**c**) Hepatic gene expression determined by real-time quantitative PCR of lysophospholid acyltransferase 3 (Lpcat3) normalized to HPRT, β-actin, and B_2_M. Results are mean ± S.E.M., n = 6–7. *P < 0.05, ***P < 0.001 vs control as determined by two-way ANOVA and Bonferronni Post Hoc tests.

We determined that CA-induced cholestasis resulted in the typical elevated hepatic lipid profile in WT mice, which was blunted in TRPC5 KO mice. We then utilised UPLC-MS to characterize the hepatic lipid content of CA-induced cholestasis. CA-feeding increased the abundance of unsaturated lysophosphatidylcholine species (LPC 16:00 and LPC 18:00) compared to control-fed mice (Fig. 4a–b); this increase in total LPC was significantly reduced in TRPC5 KO mice, suggesting enhanced clearance (Fig. 4b). Analysis of most abundant PC species, including PC38:3, PC38:4, and PC36:4 demonstrated a reduction in CA-fed mice in both groups, while PC 36:3 levels were increased in CA- WT and TRPC5 KO mice (Fig. 4a,b). However, collectively, total PC levels were not significantly different between groups (Fig. 4b).

De novo phospholipid synthesis and degradation are tightly regulated processes, involving deacylation by phospholipases and reacylation by lysophospholid acyltransferase (LPCATs)^28^. We investigated the transcriptional expression of LPCAT and found limited expression of LPCAT1 in the liver, whilst levels of LPCAT3 were abundant as previously shown^28^. Moreover, CA-feeding resulted in a significant induction in the expression of LPCAT3 in WT mice (Fig. 4c) compared to control-fed mice (P < 0.05). This induction was not observed in TRPC5 KO mice, consistent with the attenuated hepatic lipid and TG content.

The lipid sensitive profile of TRPC5^29^ prompted us to investigate its role in cholestasis, a disease characterized by elevated BAs, in addition to secondary dyslipidemia. We report here a key role for TRPC5 in a murine model of cholestasis, where we show that ablation of TRPC5 was associated with an improved outcome following chronic dietary supplementation with CA. Elevated liver enzymes, hepatic bile acid content, inflammation and dyslipidemia were significantly attenuated in TRPC5 KO mice, suggesting that TRPC5 is positively involved in CA-induced cholestasis. Plasma markers of liver injury, such as ALT, AST, ALP, GGT and albumin were substantially increased in CA-fed WT mice compared to control diet-fed mice. Furthermore, we observed significant enlargement of the liver, a hallmark of cholestasis^16^ in WT mice following CA-feeding. These processes were largely blunted in TRPC5 KO mice, suggesting a detrimental role for TRPC5 in cholestasis. Consistently, we observed high levels of bile acids (BAs), including primary and secondary bile acids, in liver samples of CA-fed WT mice, which were not detected in TRPC5 KO mice. In addition to their detergent effects, accumulation of BAs promotes hepatotoxicity via several mechanisms, including mitochondrial damage, promotion of a profibrogenic profile, and, ultimately, apoptosis and necrosis^30–32^. Overall, our results demonstrate novel findings that TRPC5 has the ability to orchestrate the development of cholestasis.

Endogenous BAs (including CA) activate FXR and its associated short heterodimer partner (SHP) and have diverse physiological roles, including regulation of lipids, glucose, and energy metabolism through modulation of gene expression^33^. We investigated the effects of chronic CA feeding on bile acid homeostasis and found no difference in the hepatic expression of FXR, which has been shown to be differentially regulated in different models of cholestasis. However, SHP mRNA expression was increased following CA-diet feeding in WT mice, and to a lesser extent in TRPC5 KO mice. Interestingly, while BA concentrations were reduced in CA-fed TRPC5 KO mice, we detected a significant repression of CYP7A1, the rate-limiting enzyme for the production of BAs from cholesterol^34^. These results highlight the complex interplay in BAs and lipid homeostasis, and suggest that, 1) CA-induced dyslipidaemia is partially dependent on TRPC5 activity, while 2) CA-induced repression of CYP7A1, known to be dependent on FXR and other factors/mediators^35^, is independent of TRPC5. This may underlie the equal repression of CYP7A1 following CA-feeding.

In line with this, CA-induced cholestasis resulted in significant deficits in lipid homeostasis as indicated by elevated levels of hepatic cholesterol and triglycerides. This dyslipidaemia may be attributed to either elevated synthesis, or defective clearance^36^. As we determined that levels of VLDL and small LDL were markedly increased following CA-diet feeding in WT mice, these results suggest enhanced synthesis. Consistent with this, we observed an induction in the mRNA expression of lipogenic genes such as SREBP1, SCD1 and FAS^37^. Hepatic excretion of lipids is dependent upon BAs formed from the catabolism of cholesterol^38, 39^. As CA-diet feeding resulted in a dysregulation of BA homeostasis, it is likely that the aberrant lipid profile observed in WT mice was secondary to these defects.

Indeed, hepatic lipid analysis revealed marked dysregulation in phosphatidylcholine (PC) and LPC, which are downstream TG metabolites^40, 41^, in addition to being involved in a wide spectrum of cellular signaling processes^30^. Crucially, biliary PC excretion is important for preventing biliary bile acid toxicity by formation of mixed micelles with BAs and cholesterol^18^, and is dependent on bile acid transporters^42^. Several PC precursors demonstrated a reduction in samples from CA-fed animals, equally in WT and TRPC5 KO mice, which may be attributed to enhanced degradation and/or generation of LPC^43, 44^. In line with this, we observed accumulation of total hepatic LPC in WT mice, which was blunted in TRPC5 KO mice. Furthermore, we observed an induction in the expression of biliary transporters, which mediate the efflux of bile acids (Bsep), and organic anions (Mpr2/ Mrp3) into bile^19, 24^ in CA-fed WT mice, which was markedly blunted in TRPC5 KO mice. LPC has documented pro-inflammatory roles in a variety of acute and chronic inflammatory diseases^45, 46^. Moreover, LPC and its precursor, lysophosphatidic acid (LPA) have been implicated in the pathogenesis of cholestasis in humans^17^, and both phospholipids have been shown to directly stimulate TRPC5^10, 29, 47^. Regulation of phospholipids is driven by a mileu of factors including LPCAT enzymes^28^. LPCAT3 belongs to the membrane-bound-*O*-acyltransferase (MBOAT) family and is primarly localized in the endoplasmic reticulum with abundant expression in metabolic tissues including liver, adipose tissue and pancreas^48^. Critically, induction of LPCAT3 was shown to be pivotal for the hepatic production of TG and VLDL^28^. Therefore, our data suggest that CA-induced cholestasis is associated with hepatic dysregulation of phospholipid metabolism, giving rise to dyslipidaemia that is TRPC5 dependent.

Collectively, the data reveals attenuated liver pathology in TRPC5 KO mice, signifying a role for TRPC5 in cholestasis-induced local liver injury. These findings provide evidence that TRPC5 activation plays a causal role in the onset and development of cholestasis-induced liver pathology and may provide a novel therapeutic target.

## Materials and Methods

### Reagents

All drugs and reagents were from Sigma-Aldrich (Dorset, U.K.) unless otherwise stated.

### Mice

Animals were housed in temperature (22 ± 2 °C)-controlled colony rooms maintained under filtered positive pressure ventilation on a 12–12 h light/dark cycle beginning at 07:00 GMT with free access to water and food. Male, age-matched 129S1/SvImJ wildtype (WT) and TRPC5 knockout (KO) littermates were used at 8–12 weeks of age. WT and TRPC5 KO mice breeding pairs were provided kindly by Prof. D.E. Clapham (Howard Hughes Medical Institute, Boston, USA)^4^. The genotype of each animal was established by PCR as previously described^4^ and as characterized by us^11^. All experiments were conducted under the guidelines of the United Kingdom Home Office Animals (Scientific Procedures) Act 1986 and were approved by the King’s College London Animal Care and Ethics Committees. Animals were randomly assigned to control or treatment groups and the experimenter was blinded towards the genetic background of animals at the time of experiment.

### Dietary induction of cholestasis

Mice were randomly allocated to receive standard animal maintenance diet (RM3, Special Diet Services, UK) or supplemented with 0.5% CA (0.5% CA in RM3 diet, Special Diet Services, UK), for 21 days as previously described^19^. Food and water consumption, and body weight were recorded every other day for the duration of the study.

### Tail-cuff Plethysmography

Blood pressure (BP) for WT and TRPC5 KO mice was measured by tail cuff plethysmography, using the CODA 8 non-invasive BP acquisition technique system for mice (Kent Scientific, Torrington, CT, USA), following a week of training. Mice were acclimatised to the room for 1 h and allowed to acclimatise to the restraint tubes for 10 min before the recording of BP^49^. Results of 15 measurements were taken every other day from diet supplementation for 21 days.

### Measurement of neutrophil accumulation

A spectrophotometric assay was used to determine myeloperoxidase (MPO) activity, as described previously^22^. Liver samples were homogenized in the presence of hexadecyltrimethylammonium bromide (HTAB) in order to disrupt the granules, and centrifuged at 10,000 × *g* for 15 min. MPO activity was then analysed, and normalized to protein content.

### Plasma/liver biochemistry

Mice were anaesthetised with isoflurane (2–3% carried in 2–3% O_2_) and blood samples were obtained via a cardiac puncture, using a heparinised syringe and needle (100 U/ml). Plasma was separated with centrifugation (400 × *g*, 20 min), snap frozen in liquid nitrogen and stored at −80 °C until processing. Whole liver was weighed and snap frozen in liquid nitrogen for biochemical assays. Plasma and hepatic triglyceride (TG) (Cayman, UK) and total cholesterol (Abcam, UK) were quantified as per manufacturer’s instructions. Plasma aspartate aminotransferase (AST), alanine aminotransferase (ALT), alkaline phosphatase (ALP), and gamma glutamyltransferase (GGT) levels were determined following manufactuer’s instructions (Sigma-Aldrich, UK). Plasma albumin was measured according to manufacturer’s instructions (Abcam, UK). Liver samples were homogenised in 0.125 M potassium phosphate buffer with protease inhibitors, as previously described^19^; results were normalised to protein content.

### Bile and lipidomic analysis by LC-MS

Snap frozen mice liver biopsies (50–80 mg) were used for separate extractions of bile acids and lipids^50, 51^. Analysis of extracted samples were performed using Waters ACQUITY ultra-performance liquid chromatography-quadrupole time of flight (UPLC-QToF) in positive ionisation mode for lipids and negative ionisation mode for bile acids. All lipids were measured using the Waters MassLynx software (Waters Corporation, Milford, MA) and peak areas were normalized to internal standards. The identification was performed by comparing structure and fragmentation patterns in the MS^2^ data with standards; results are expressed as normalised area.

### Quantitative Polymerase Chain Reaction (qPCR)

Approximately 50 mg of liver tissue was dissected and immersed in *RNALater* RNA Stabilisation Reagent (Qiagen, UK) before being stored at −20 °C. RNA was extracted using the RNEasy Microarray kit according to manufacturer’s instructions (Qiagen). First-strand complementary DNA (cDNA) was synthesised from 1000 ng of total RNA using the High-Capacity RNA-cDNA kit supplemented with RNAse inhibitors according to manufacturer’s instructions (Applied Biosystems, UK)^52^. Primers were designed in-house using Primer Blast software (http://www.ncbi.nlm.nih.gov/tools/primer-blast/), and synthesised by Sigma; details of primers and PCR product sizes are listed in Supplementary Table 1. Quantitative PCR (qPCR) was performed with the SensiMix SYBR No-ROX Kit (Bioline, London, United Kingdom) with Hot-Start Taq polymerase on a Corbett Rotorgene 6000 (Qiagen, UK). Samples were heated to 95 °C for 10 min (initial denaturation), followed by 45 cycles of 10 s at 95 °C, 15 s at 57 °C, and 5 s at 72 °C; melt at 68–90 °C with fluorescence detection after each cycle. Samples were subjected to melting curve analysis to confirm product specificity. Expression of each gene was analysed as copy/µl derived from a standard curve using the Rotorgene 6000 software. Results were normalised to geometric mean of hypoxanthine guanine phosphoribosyl transferase (HPRT), β-actin and β_2_ microglobulin (B_2_M) using GeNorm version 3.4^53^.

### Statistical Analysis

Results are presented as mean ± SEM. Statistical differences between samples were determined by two-way analysis of variance (ANOVA) and Bonferroni *post hoc* test using GraphPad Prism (GraphPad Prism, V 5.02 for Windows, GraphPad Software, La Jolla California, USA). *P* < 0.05 was considered to represent a significant difference.

## Electronic supplementary material

Supplemental figures

## References

[1] Montell C, Birnbaumer L, Flockerzi V (2002). The TRP channels, a remarkably functional family. Cell.

[2] Bon RS, Beech DJ (2013). In pursuit of small molecule chemistry for calcium-permeable non-selective TRPC channels – mirage or pot of gold?. Br J Pharmacol.

[3] Clapham DE (2003). TRP channels as cellular sensors. Nature.

[4] Riccio A (2009). Essential role for TRPC5 in amygdala function and fear-related behavior. Cell.

[5] Beech DJ (2007). Bipolar phospholipid sensing by TRPC5 calcium channel. Biochem Soc Trans.

[6] Zholos AV (2014). Trpc5. Handb Exp Pharmacol.

[7] Edwards JM (2010). Exercise training decreases store-operated Ca2+ entry associated with metabolic syndrome and coronary atherosclerosis. Cardiovascular research.

[8] Kumar B (2006). Upregulated TRPC1 channel in vascular injury *in vivo* and its role in human neointimal hyperplasia. Circulation research.

[9] Sukumar P (2012). Constitutively active TRPC channels of adipocytes confer a mechanism for sensing dietary fatty acids and regulating adiponectin. Circ Res.

[10] Flemming PK (2006). Sensing of lysophospholipids by TRPC5 calcium channel. The Journal of biological chemistry.

[11] Alawi, K. M. *et al*. Transient receptor potential canonical 5 (TRPC5) protects against pain and vascular inflammation in arthritis and joint inflammation. *Ann Rheum Dis*, doi:10.1136/annrheumdis-2015-208886 (2016). 10.1136/annrheumdis-2015-208886 PMC5264234 27165180

[12] El Boustany C (2008). Capacitative calcium entry and transient receptor potential canonical 6 expression control human hepatoma cell proliferation. Hepatology.

[13] Wang J (2006). Hypoxia inducible factor 1 mediates hypoxia-induced TRPC expression and elevated intracellular Ca2+ in pulmonary arterial smooth muscle cells. Circulation research.

[14] Xu SZ, Beech DJ (2001). TrpC1 is a membrane-spanning subunit of store-operated Ca(2+) channels in native vascular smooth muscle cells. Circulation research.

[15] Saleh SN, Albert AP, Peppiatt-Wildman CM, Large WA (2008). Diverse properties of store-operated TRPC channels activated by protein kinase C in vascular myocytes. The Journal of physiology.

[16] Yeh TH (2010). Liver-specific beta-catenin knockout mice have bile canalicular abnormalities, bile secretory defect, and intrahepatic cholestasis. Hepatology.

[17] Kremer, A. E. *et al*. Lysophosphatidic acid is a potential mediator of cholestatic pruritus. *Gastroenterology* **139**, 1008–1018, 1018 e1001, doi:10.1053/j.gastro.2010.05.009 (2010). 10.1053/j.gastro.2010.05.009 20546739

[18] Baghdasaryan A (2008). Role of hepatic phospholipids in development of liver injury in Mdr2 (Abcb4) knockout mice. Liver Int.

[19] Papacleovoulou G (2013). Maternal cholestasis during pregnancy programs metabolic disease in offspring. J Clin Invest.

[20] Modica, S. *et al*. Selective activation of nuclear bile acid receptor FXR in the intestine protects mice against cholestasis. *Gastroenterology* **142**, 355–365, e351–354, doi:10.1053/j.gastro.2011.10.028 (2012). 10.1053/j.gastro.2011.10.028 22057115

[21] Fuchs TA, Kremer Hovinga JA, Schatzberg D, Wagner DD, Lammle B (2012). Circulating DNA and myeloperoxidase indicate disease activity in patients with thrombotic microangiopathies. Blood.

[22] Keeble J (2005). Involvement of transient receptor potential vanilloid 1 in the vascular and hyperalgesic components of joint inflammation. Arthritis and rheumatism.

[23] Wang R (2003). Severe cholestasis induced by cholic acid feeding in knockout mice of sister of P-glycoprotein. Hepatology.

[24] Abu-Hayyeh S (2013). Intrahepatic cholestasis of pregnancy levels of sulfated progesterone metabolites inhibit farnesoid X receptor resulting in a cholestatic phenotype. Hepatology.

[25] Claudel T (2002). Bile acid-activated nuclear receptor FXR suppresses apolipoprotein A-I transcription via a negative FXR response element. The Journal of clinical investigation.

[26] Goodwin B (2000). A regulatory cascade of the nuclear receptors FXR, SHP-1, and LRH-1 represses bile acid biosynthesis. Mol Cell.

[27] Xu G (1996). Increasing hepatic cholesterol 7alpha-hydroxylase reduces plasma cholesterol concentrations in normocholesterolemic and hypercholesterolemic rabbits. Hepatology.

[28] Rong, X. *et al*. Lpcat3-dependent production of arachidonoyl phospholipids is a key determinant of triglyceride secretion. *eLife* **4**, doi:10.7554/eLife.06557 (2015). 10.7554/eLife.06557 PMC4400582 25806685

[29] Beech DJ, Bahnasi YM, Dedman AM, Al-Shawaf E (2009). TRPC channel lipid specificity and mechanisms of lipid regulation. Cell Calcium.

[30] Beyoglu D, Idle JR (2013). The metabolomic window into hepatobiliary disease. Journal of hepatology.

[31] Rudraiah S, Zhang X, Wang L (2016). Nuclear Receptors as Therapeutic Targets in Liver Disease: Are We There Yet?. Annual review of pharmacology and toxicology.

[32] Wagner M, Zollner G, Trauner M (2011). Nuclear receptors in liver disease. Hepatology.

[33] Lefebvre P, Cariou B, Lien F, Kuipers F, Staels B (2009). Role of bile acids and bile acid receptors in metabolic regulation. Physiol Rev.

[34] Jelinek DF, Andersson S, Slaughter CA, Russell DW (1990). Cloning and regulation of cholesterol 7 alpha-hydroxylase, the rate-limiting enzyme in bile acid biosynthesis. J Biol Chem.

[35] Sinal CJ (2000). Targeted disruption of the nuclear receptor FXR/BAR impairs bile acid and lipid homeostasis. Cell.

[36] Moreno-Navarrete JM (2012). The L-alpha-lysophosphatidylinositol/GPR55 system and its potential role in human obesity. Diabetes.

[37] Schmitt J (2015). Protective effects of farnesoid X receptor (FXR) on hepatic lipid accumulation are mediated by hepatic FXR and independent of intestinal FGF15 signal. Liver international: official journal of the International Association for the Study of the Liver.

[38] Vlahcevic ZR, Pandak WM, Hylemon PB, Heuman DM (1993). Role of newly synthesized cholesterol or its metabolites on the regulation of bile acid biosynthesis after short-term biliary diversion in the rat. Hepatology.

[39] Vlahcevic, Z. R., Pandak, W. M. & Stravitz, R. T. Regulation of bile acid biosynthesis. *Gastroenterology clinics of North America* **28**, 1–25, v (1999). 10.1016/s0889-8553(05)70041-8 10198776

[40] Cases S (1998). Identification of a gene encoding an acyl CoA:diacylglycerol acyltransferase, a key enzyme in triacylglycerol synthesis. Proceedings of the National Academy of Sciences of the United States of America.

[41] Xu J (2015). Lipidomics comparing DCD and DBD liver allografts uncovers lysophospholipids elevated in recipients undergoing early allograft dysfunction. Scientific reports.

[42] Oude Elferink RP (1995). Regulation of biliary lipid secretion by mdr2 P-glycoprotein in the mouse. The Journal of clinical investigation.

[43] Kume N, Cybulsky MI, Gimbrone MA (1992). Lysophosphatidylcholine, a component of atherogenic lipoproteins, induces mononuclear leukocyte adhesion molecules in cultured human and rabbit arterial endothelial cells. The Journal of clinical investigation.

[44] Quinn MT, Parthasarathy S, Fong LG, Steinberg D (1987). Oxidatively modified low density lipoproteins: a potential role in recruitment and retention of monocyte/macrophages during atherogenesis. Proceedings of the National Academy of Sciences of the United States of America.

[45] Goncalves I (2012). Evidence supporting a key role of Lp-PLA2-generated lysophosphatidylcholine in human atherosclerotic plaque inflammation. Arteriosclerosis, thrombosis, and vascular biology.

[46] Orosa B, Garcia S, Conde C (2015). The autotaxin-lysophosphatidic acid pathway in pathogenesis of rheumatoid arthritis. European journal of pharmacology.

[47] E AL-S (2011). GVI phospholipase A2 role in the stimulatory effect of sphingosine-1-phosphate on TRPC5 cationic channels. Cell calcium.

[48] Zhao Y (2008). Identification and characterization of a major liver lysophosphatidylcholine acyltransferase. The Journal of biological chemistry.

[49] Marshall NJ (2013). A role for TRPV1 in influencing the onset of cardiovascular disease in obesity. Hypertension.

[50] Legido-Quigley C (2011). Bile UPLC-MS fingerprinting and bile acid fluxes during human liver transplantation. Electrophoresis.

[51] Whiley L, Godzien J, Ruperez FJ, Legido-Quigley C, Barbas C (2012). In-vial dual extraction for direct LC-MS analysis of plasma for comprehensive and highly reproducible metabolic fingerprinting. Analytical chemistry.

[52] Alawi, K. M. *et al*. The sympathetic nervous system is controlled by transient receptor potential vanilloid 1 in the regulation of body temperature. *FASEB J*, doi:10.1096/fj.15-272526 (2015). 10.1096/fj.15-272526 PMC4650996 26136480

[53] Vandesompele, J. *et al*. Accurate normalization of real-time quantitative RT-PCR data by geometric averaging of multiple internal control genes. *Genome Biol* **3**, RESEARCH0034 (2002). 10.1186/gb-2002-3-7-research0034 PMC126239 12184808

