# Supplementary material for: Transient receptor potential canonical 5 channels plays an essential role in hepatic dyslipidemia associated with cholestasis
Source: Sci Rep. 2017 May 24;7:2338. doi: 10.1038/s41598-017-02439-z (PMC5443755; doi:10.1038/s41598-017-02439-z)
Supplement: Supplementary file 1 — Supplemental figures [file 41598_2017_2439_MOESM1_ESM.docx]

**Supplementary Figures**

**Transient receptor potential canonical 5 channels plays an essential role in hepatic dyslipidemia associated with cholestasis**

Khadija M. Alawi^1*^, David Tandio^1*^, Jin Xu^2^, Pratish Thakore^1,2^, Georgia Papacleovoulou^3^, Elizabeth S. Fernandes^1,4^, Cristina Legido-Quigley^2^, Catherine Williamson^3^, & Susan D. Brain^1^.

^1^BHF Cardiovascular Centre of Excellence and Centre of Integrative Biomedicine, Cardiovascular Division, King’s College London, London, UK, ^2^Institute of Pharmaceutical Sciences, Faculty of Life Sciences &Medicine, King’s College London, London, UK, ^3^Division of Women’s Health, Women’s Health Academic Centre, King’s College London, London, UK, ^4^ Programa de Pós-Graduação, Universidade Ceuma, São Luís, Brazil.

^*^ Denotes joint first authors


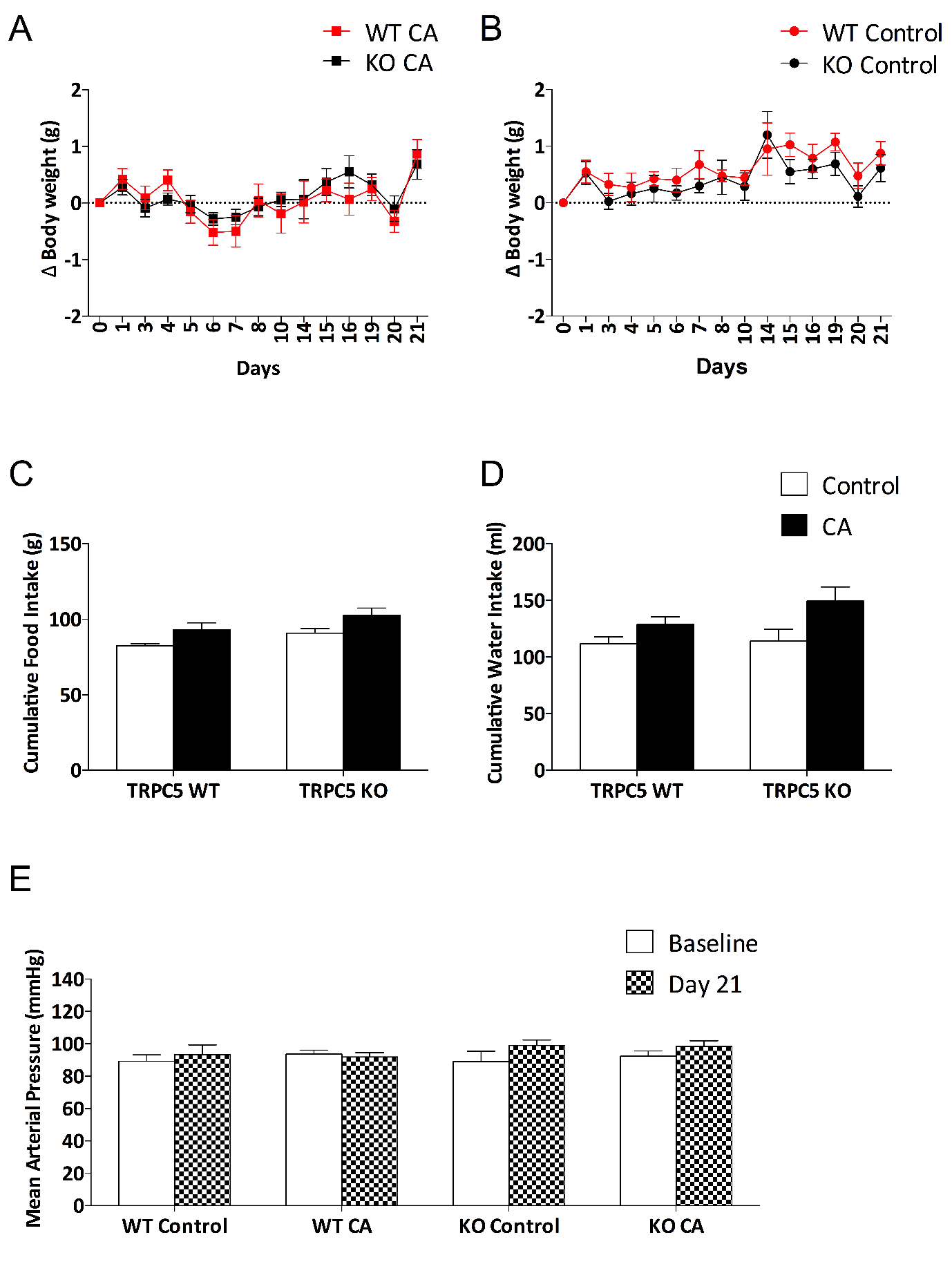


**Supplementary Figure 1.** ***In vivo* profile of CA-induced cholestasis.** (A-B) Time-course of relative body weight WT and TRPC5 KO mice maintained on 0.5% CA diet, or control (RM3) diet. Cumulative food (C), and water (D) intake over 21 days. (E) Mean arterial pressure at baseline and 21 days following control (RM3) or 0.5% CA diet feeding in WT and TRPC5 KO mice. Results are mean ± S.E.M., n=9-10.


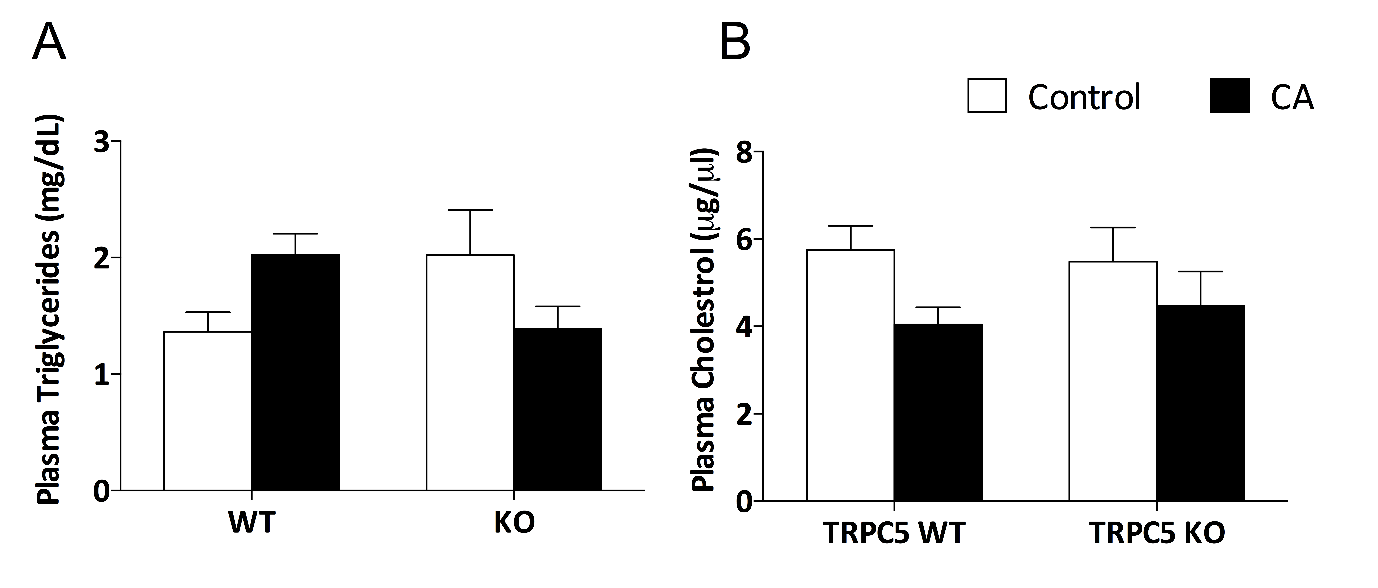


**Supplementary Figure 2.** **Plasma lipid profile following CA-induced cholestasis.** (A) Plasma triglyceride (TG) and (B) cholesterol levels were determined in WT and TRPC5 KO mice 21 days following chow- or CA-diet feeding. Results are mean ± S.E.M., n=6-7.

**Supplementary Figure 3.** Representative Haematoxylin and Eosin staining of paraffin-embedded liver sections of WT and TRPC5 KO mice 21 days following control or 0.5% CA diet feeding. Scale bar represents 100µm.

| ***Gene symbol*** | ***Primer Sequence*** | ***Accession number*** |
| --- | --- | --- |
| *Abcb11* | F: TCACATCTGTAGGGTTGTTGAGT  R: GAGCAATGCGCACACACTTC | NM_021022.3 |
| *Abcc2* | F: TGCCCTGGAAATCACGATGG  R: AAGCCCAAGGGAATCCACAC | NM_013806.2 |
| *Abcc3* | F: GCAGCAGAACCAAGCATCAAG  R: GACCGCATCCTCACCTGG | NM_029600.3 |
| *Actb* | F: CACAGCTTCTTTGCAGCTCCTT  R: TCAGGATACCTCTCTTGCTCT | NM_007393.5 |
| *Apoe* | F: CAGACCCTGGAGGCTAAGGA  R: TCGGCTAGGCATCCTGTCA | NM_009696.4 |
| *B2m* | F: GTCGCTTCAGTCGTCAGCA  R: TTGAGGGGTTTTCTGGATAGCA | NM_009735.3 |
| *Cyp7a1* | F: CTGGGGGATTGCTGTGGTAG  R: GGTTCACCTACTTTCCTTCTCCT | NM_007824.2 |
| *Fasn* | F: TGACTCGGCTACTGACACGA  R: GTGGTGGAACCCTCAATGGG | NM_007988.3 |
| *Hprt* | F: TCCTCCTCAGACCGCTTT T  R: CCTGGTTCATCATCGCTAATC | NM_013556.2 |
| *Lpcat3* | F: ATCTTCCTGGGCTACCCGTT  R: ACTGAAGCACGACACATAGCA | NM_145130.2 |
| *Nr0b2* | F: CCTGGAGTCTTTCTGGAGCCT  R: GGCACATCTGGGTTGAAGAGGAT | NM_011850.2 |
| *Nr1h4* | F: TCTCAGTTGCCGTGAGGAAG  R: GCGTGTTCTGTTAGCATACCTTT | NM_001163700.1 |
| *Scd1* | F: GAGTACGTCTGGAGGAACATCA  R: GTCATGTAGTAGAAAATCCCGAAG | NM_009127.4 |
| *Srebf1* | F: GTCACCGTTTCTTTGTGGACG  R: GAACTCCCTGTCTCCGTCA | NM_011480.4 |

**Supplementary Table 1. Mouse primer sequences used in qPCR studies.** F: Forward, R: Reverse. Abcb11; ATP-binding cassette, sub-family B (MDR/TAP), member 11, Abcc2; ATP-binding cassette, sub-family C (CFTR/MRP), member 2, Abcc3; ATP-binding cassette, sub-family C (CFTR/MRP), member 3, Actb; beta actin, Apoe; apolipoprotein E, B2m; beta-2 microglobulin, Cyp7a1; cytochrome P450, family 7, subfamily a, polypeptide 1, Fasn; fatty acid synthase, Hprt; hypoxanthine guanine phosphoribosyl transferase, Lpcat3; lysophosphatidylcholine acyltransferase 3, Nr0b2; nuclear receptor subfamily 0, group B, member 2, Nr1h4; nuclear receptor subfamily 1, group H, member 4, Scd1; stearoyl-Coenzyme A desaturase 1, Srbf1; sterol regulatory element binding transcription factor 1.
